# Observations on Bromide of Ethyl in Dental Surgery

**Published:** 1891-06

**Authors:** J. F. W. Silk


ARTICLE II.

### OBSERVATIONS ON BROMIDE OF ETHYL IN DENTAL SURGERY.

Dr. J. F. W. Silk, M. D., read a paper before the Odontological Society of Great Britain as follows:

In America, on the Continent, and especially in Germany, the drug has been held in high repute by dental surgeons for some time past, but in this country there were no records of its systematic employment in a similar manner. At the outside, Dr. Silk wished to disclaim any advocacy of the indiscriminate use of the bromide or any suggestion that it was an entirely satisfactory substitute for the other dental anæsthetics. He had endeavored to impartially record his table of cases, and he desired to be equally impartial in his remarks, so that each might form his own opinion as to whether the drug was worthy of a more extended trial. Bromide of ethyl seemed first to have been used as an anæsthetic by Nunneley, of Leeds, in 1849, and again brought forward by him in 1865; some years later its use was advocated by Turnbull, and by Levis in America. For its introduction into dental work, Dr. Silk believed Schneider of Berlin, and Hertz of Vienna were responsible. As to its properties, without entering minutely into the subject, care should be taken that when bromide of ethyl ($C_2 H_5 Br$) was asked for, the chemist did not supply bromide of ethylene ($C_2 H_4 Br.$), the substitution of which had led to fatal results. Merck's preparation was almost universally used in Germany, and notwithstanding a leading English chemist's assurance of the purity of some English preparations, Dr. Silk strongly favored Merck's. Variation in effect might be partly or even entirely due to the decomposition which the drug undergoes by keeping. As to the cost, where any number of administrations are undertaken, it might be said to be very little more than half the cost of nitrous oxide per patient. It would seem that robust and healthy males tolerate the drug better than weakly anæmic females, or children. The amount of the dose would vary, *inter alia*, with the method of administration, *e. g.*, in the first seven cases with the open or semi-open method *m*70 to ʒ 3 were required. With the closed method ʒ iss had usually proved ample, and in many instances ʒ i had sufficed. He would be inclined to put the dose down at from ʒ i to ʒ iss. Being not altogether satisfied with the inhaler used in the first seven cases, Dr. Silk adopted an ordinary Ormsby's inhaler, upon which the drug is poured through the face-piece on to the sponge, and it is then fitted to the face, little or no air is admitted for the first few inhalations, after which the cap of the air-hole is slightly turned. The induction of anæsthesia with this inhaler averaged 66.8 seconds, and the duration 46.2 seconds—half as long again as with nitrous oxide. The average of induction was based upon the observation of twenty, and the average of duration upon twenty-eight cases.

Roughly, the duration of the anæsthesia bears some relation to the time required for induction, but this is by no means absolute, and with the English preparation of the drug the variability is very marked. When the time from the commencement of the inhalation to the end of the anæsthesia exceeds two minutes, the after-effects are likely to be bad. The question, "How do you know where a proper degree of anæsthesia has been produced ?" is not so simply answered as might seem. In Berlin some stress seemed to be laid upon the muscular relaxation, but Dr. Silk had not found this at all reliable. He generally removed the face-piece when the faintest stertor was heard, or if that were obviously due to accidental causes or unduly delayed, the commencing weakness of the pulse or the development of conjunctival insensibility were his guides. The stertor referred to is not the laryngeal stertor of nitrous oxide narcosis, but of a much lighter and regular kind, originating probably in the palate. During the course of the inhalation it would probably be found that the breathing becomes slightly slower and shallower, but in the majority of cases these changes are almost inappreciable. Dr. Silk had never been able to detect any irregularity in the rhythm, nor, as a general rule, had any cough, laryngeal spasm, or bronchial irritation been set up. These remarks apply only to the methods of dental anæsthesia, and were not applicable to the effects produced when the anæsthesia is prolonged for longer periods. There could be no doubt, in his opinion, that the cardio-vascular or circulatory system is much disturbed during the inhalation of bromide of ethyl. The primary flushing of the face is, he believed, a pretty constant phenomenon, though it might be but momentary in its duration; this effect is very similar to that produced by nitrite of amyl, and might possibly be explained in the same way, *i. e.*, arterial dilatation with consequent fall of blood pressure. This fall might, no doubt, to a certain extent, in itself account for the diminution in force and increased frequency of the heart-beat as observed at the radial pulse. It was, however, highly probable that other factors are at work, for the flushing quickly succeeds a pallor and the heart's action tends to become more feeble, slower and irregular.

Dr. Silk thought upon the whole that his sphymographic tracings (exhibited) bore out the views he had put forward in respect to the effects of the bromide on the circulatory system, especially if it be remembered that the heart's action immediately before the inhalation is probably unduly accelerated by the nervous condition of the patient. One set of tracings he regarded as particularly interesting; as indicating the rapidity with which the blood pressure, etc., is restored. This rapid recovery is, he thought, usual and is of great importance.

As to the effect of the drug upon the nervous system, and through it upon the muscular system, the exceedingly transient character of the stage of excitement was striking, in the majority of cases it had been almost inappreciable. This observation seemed to apply almost equally to the nervous woman, and the robust (and probably alcoholic) navvy. It might possibly serve as an indication for the class of cases in which the use of the drug is more particularly appropriate. Occasionally very slight spasm of the fingers, or rhythmic movement of the limbs, etc., were exhibited, but Dr. Silk had never observed anything like the degree of spasm and movement which often accompanies nitrous oxide or ether. In respect to other phenomena he had not much to say. The pupils tend to dilate. Increase in the salivary secretions was sufficiently marked in four cases to attract attention, but never so copious as with ether. In some few instances, patients would make a groaning noise from the beginning of the inhalation, but this had always been ignored and did not either delay or shorten the narcosis. The anæsthesia which had resulted from the use of the drug, had, to Dr. Silk's mind, always been quite satisfactory both in degree and in kind.

After-effects might be divided into immediate and remote: of the former Dr. Silk had definite notes in thirty-nine instances. In eleven, a certain amount of hysteria resulted; in ten others, more or less depression or prostration occurred. In one, actual fainting ensued, but the patient was subject to what she termed "fainting fits." Actual sickness was noted in two cases, and in seven others nausea was complained of. Slight and transient degrees of exhilaration were developed in three instances, and in many cases a certain amount of confusion of the intellect had remained for some few minutes. By the use of printed post-cards, rough estimates of remote effects had been obtained in forty-five instances. Of these forty-five, in no fewer than twenty-nine the return was to the effect that the patients were perfectly well on their return home, and for the remainder of the day. Prostration or depression was noted in five instances, vomiting in two, and headache in three. In one case the return was "ill all day," but the nature of the illness Dr. Silk was not able to ascertain, possibly it might have been simple hysteria. Of the remaining five cases, dizziness, drowsiness, and other very trivial and doubtful complaints were recorded. As to the relation between immediate and remote effects, from seventeen tabulated observations of both, it was gathered that the importance and severity of the remote symptoms were tolerably proportionate with the immediate after-effects. Thus simple hysteria had seldom been followed by anything worse, while on the other hand, nausea might be followed by sickness, or the sickness might be prolonged. This somewhat negative evidence was of importance, for it enabled Dr. Silk to say, so far as his own limited experience was concerned, that he had never met with a case in which serious symptoms had developed upwards of twelve hours after the bromide had been inhaled. Such cases had, however, been described. With reference to the death rate and dangers of bromide of ethyl, while aware of the importance of the point he could not help thinking that the fatalities which had been hitherto recorded rather tended to exaggerate the danger of the drug when used in dental surgery in the way he had described.

The editor of the *London Dental Journal* comments as follows: Probably everyone is pleased to learn that our experts are still busy experimenting with anæsthetics. The results given at the last meeting of the Odontological Society were not such as will, we imagine, encourage the adoption of Nunneley's anæsthetic among English dentists. Dr. Silk who introduced the interesting young stranger, was not able to state a very strong case for him, his appendix showed that in a rather large number of cases more or less untoward symptoms followed its use. However, it must be remember that some of these were hardly test cases, inasmuch as Dr. Silk had adopted a better method in the latter ones. The surgeons who spoke of their experience in operating upon patients who were under the bromide did not find it satisfactory, as there seemed some doubt when the patient was "under," and the after sickness, as with chloroform and ether, struck them as placing the anæsthetic behind our old familiar friend "the gas." It is curious how nitrous oxide holds its own, at one time cocaine, at another oxygen, and other gases crop up, and now bromide of ethyl, but like Tennyson's brook—"gas" 'goes on for ever.' The late Mr. Clover thought ethidene dichloride was to oust gas and everything else, but his vaticination has fallen to the ground. The anæsthetists who spoke on Dr. Silk's paper, although several of them had not tried bromide of ethyl in dental practice, spoke strongly against its use, and if as was urged by Dr. Dudley Buxton, the action of this anæsthetic is physiologically that of chloroform, its employment in dentistry must be open to the objections usually advanced against that substance.

